# Preoperative Metyrosine Administration for Bilateral Pheochromocytomas Following Pheochromocytoma Crisis: A Case Report

**DOI:** 10.1002/iju5.70267

**Published:** 2026-09-10

**Authors:** Nanaho Demizu, Daiji Takamoto, Tosihkazu Koike, Takeaki Noguchi, Masanobu Yamazaki, Akihito Hashizume, Daiki Ueno, Takashi Kawahara, Junichi Teranishi, Kazuhide Makiyama

**Affiliations:** ^1^ Department of Urology and Renal Transplantation Yokohama City University Medical Center Yokohama Japan; ^2^ Department of Urology Yokohama City University Graduate School of Medicine Yokohama Japan

## Abstract

**Introduction:**

Pheochromocytoma crisis complicated by catecholamine‐induced cardiomyopathy is a life‐threatening condition requiring careful preoperative management. Although metyrosine has recently been approved in Japan for the perioperative management of pheochromocytoma, clinical experience remains limited.

**Case Presentation:**

A 46‐year‐old man presented with pheochromocytoma crisis complicated by reverse Takotsubo cardiomyopathy and was diagnosed with bilateral pheochromocytomas. Given the preceding life‐threatening crisis, severe cardiac dysfunction, marked catecholamine excess, and bilateral disease, he was considered at high perioperative risk. Metyrosine was added to conventional α‐adrenergic blockade before one‐stage bilateral adrenalectomy. Metyrosine was well tolerated, and total urinary metanephrine decreased by approximately 76% without symptomatic hypotension. Although intraoperative blood pressure fluctuations still occurred, bilateral adrenalectomy was completed successfully.

**Conclusion:**

This case supports the feasibility of preoperative metyrosine administration in a high‐risk patient. Although its effect on intraoperative hemodynamic stability remains uncertain, metyrosine may be considered as an adjunct to conventional α‐adrenergic blockade in carefully selected patients.

## Keynote Message

Preoperative metyrosine achieved marked biochemical suppression of catecholamine production without clinically significant adverse effects in a high‐risk patient with bilateral pheochromocytomas. Although intraoperative hemodynamic fluctuations were not completely prevented, this case suggests that metyrosine can be considered as an adjunct to conventional α‐adrenergic blockade in carefully selected high‐risk patients.

## 1 Introduction

Pheochromocytoma is a catecholamine‐producing neuroendocrine tumor that may cause life‐threatening pheochromocytoma crisis, accompanied by acute heart failure, shock, or multiorgan dysfunction [1]. Catecholamine‐induced cardiomyopathy, including reverse Takotsubo cardiomyopathy, further complicates perioperative management because of severe hemodynamic instability [2]. Surgical resection is the definitive treatment, and adequate preoperative α‐adrenergic blockade is recommended [1]. Metyrosine, an inhibitor of catecholamine synthesis, has recently been approved in Japan for the perioperative management of pheochromocytoma and paraganglioma, although clinical experience in high‐risk patients remains limited [3, 4]. We report a case of bilateral pheochromocytomas diagnosed following a pheochromocytoma crisis that was managed with preoperative metyrosine before one‐stage bilateral adrenalectomy.

## 2 Case Presentation

**FIGURE 1:**
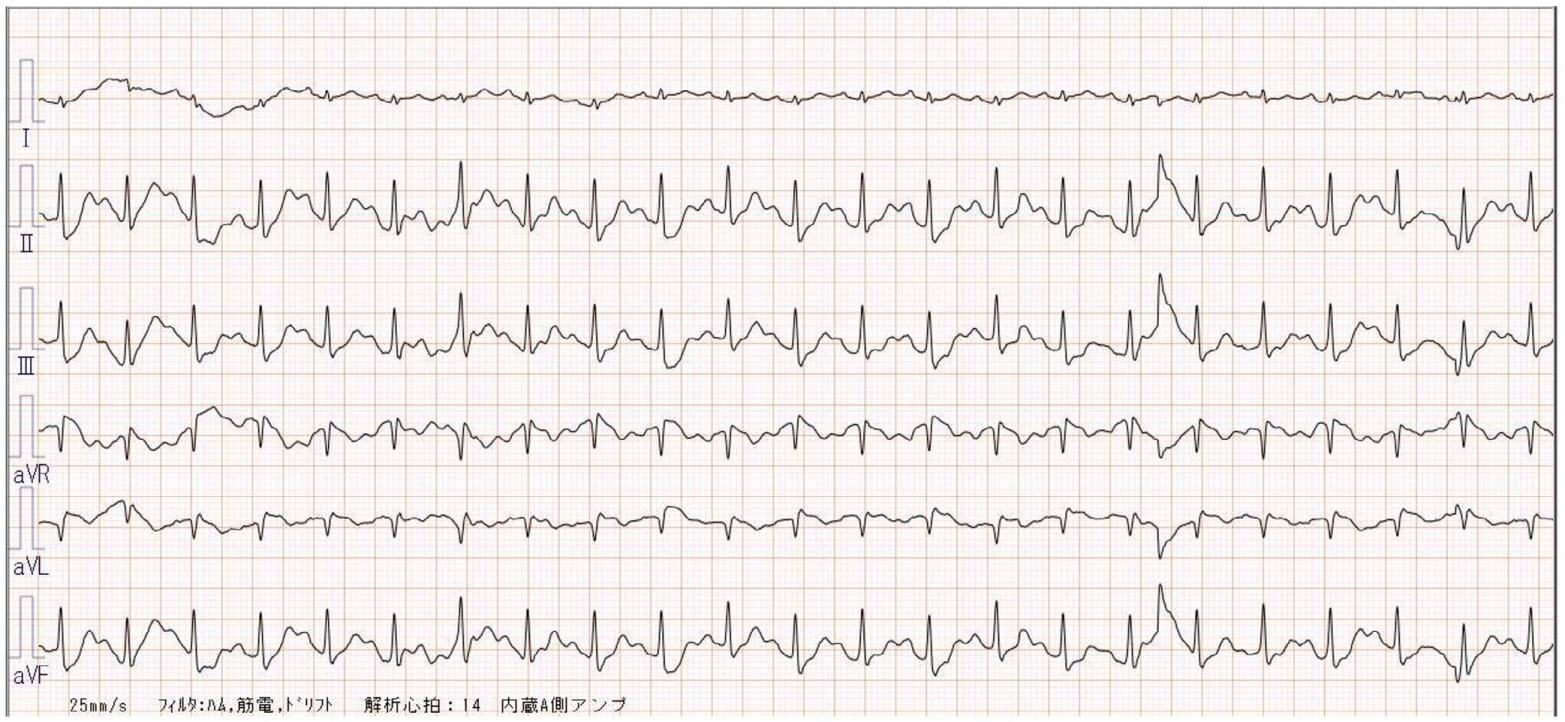
Electrocardiogram on admission. Electrocardiography demonstrated ST‐segment elevation and abnormal Q waves in leads V2–V3.

A 46‐year‐old man with no significant medical or family history presented with chest pain and dyspnea after nausea following alcohol consumption. On admission, his blood pressure was 168/115 mmHg and heart rate 141 beats/min. Electrocardiography showed ST‐segment elevation and abnormal Q waves in leads V2–V3 (Figure 1). Echocardiography revealed basal left ventricular hypokinesis with an LVEF of 20%, leading to the diagnosis of reverse Takotsubo cardiomyopathy. Severe hypoxemia required mechanical ventilation.

**FIGURE 2:**
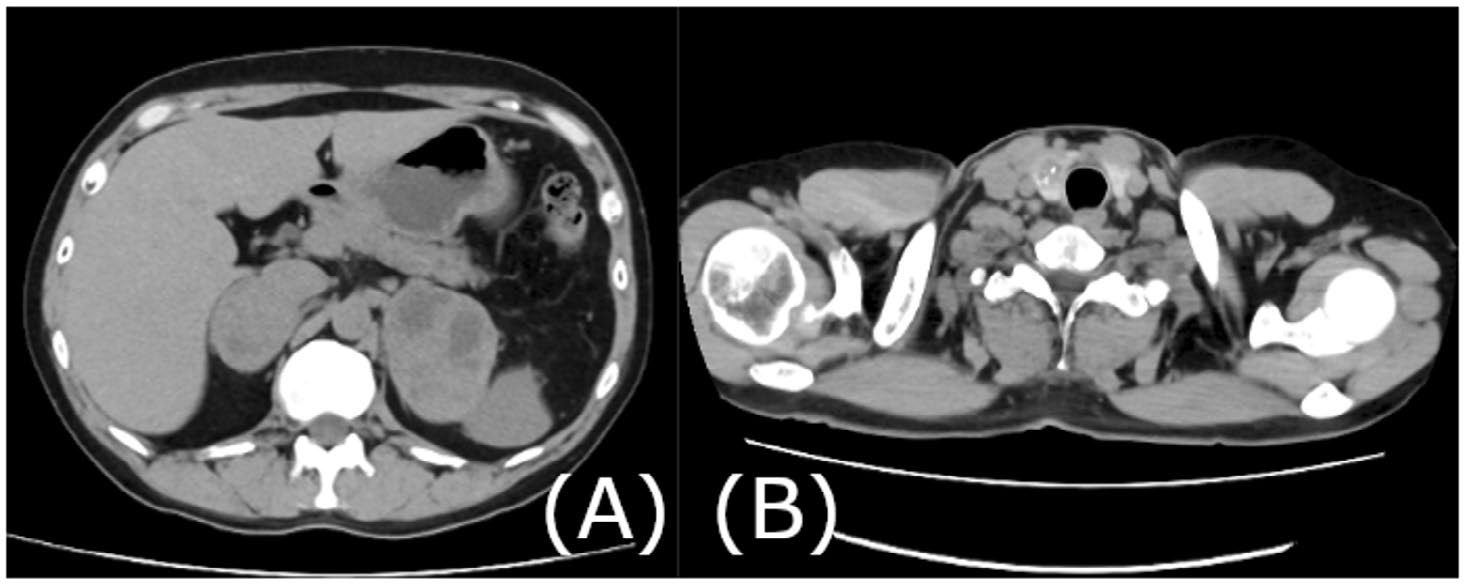
Computed tomography findings. (A) Plain computed tomography demonstrating bilateral adrenal tumors measuring 4.9 cm on the right and 7.7 cm on the left. (B) Plain computed tomography demonstrating a thyroid nodule posterior to the right thyroid lobe.

Computed tomography demonstrated bilateral adrenal tumors measuring 4.9 cm on the right and 7.7 cm on the left (Figure 2A), and a thyroid nodule (Figure 2B). Pheochromocytoma crisis was suspected based on the bilateral adrenal tumors, severe hypertension, tachycardia, cardiac dysfunction, and respiratory failure. Nicardipine was initiated, followed by doxazosin and bisoprolol. His condition improved, allowing extubation on Day 10 and discharge on Day 17.

Endocrine evaluation demonstrated marked catecholamine excess (Table 1), and ^123I‐MIBG scintigraphy showed intense bilateral adrenal uptake. Fine‐needle aspiration cytology of the thyroid nodule revealed medullary thyroid carcinoma. MEN2A was therefore strongly suspected despite the absence of a family history. RET genetic testing was performed before surgery, but the result was not yet available.

**TABLE 1:** Changes in biochemical and cardiac parameters before and after preoperative metyrosine administration.

Variable	At admission	Before surgery	After surgery
LVEF(%)	20	64.8	—
Plasma adrenaline (pg/mL)	9671	—	26
Plasma noradrenaline (pg/mL)	54 302	—	190
Plasma dopamine (pg/mL)	632	—	9
**Total urinary metanephrines (mg/day)**	**20.4**	**4.86 (−76%)**	**0.43**
Urinary metanephrine (mg/day)	8.2	2.42	0.16
Urinary normetanephrine (mg/day)	12.2	2.44	0.27

**FIGURE 3:**
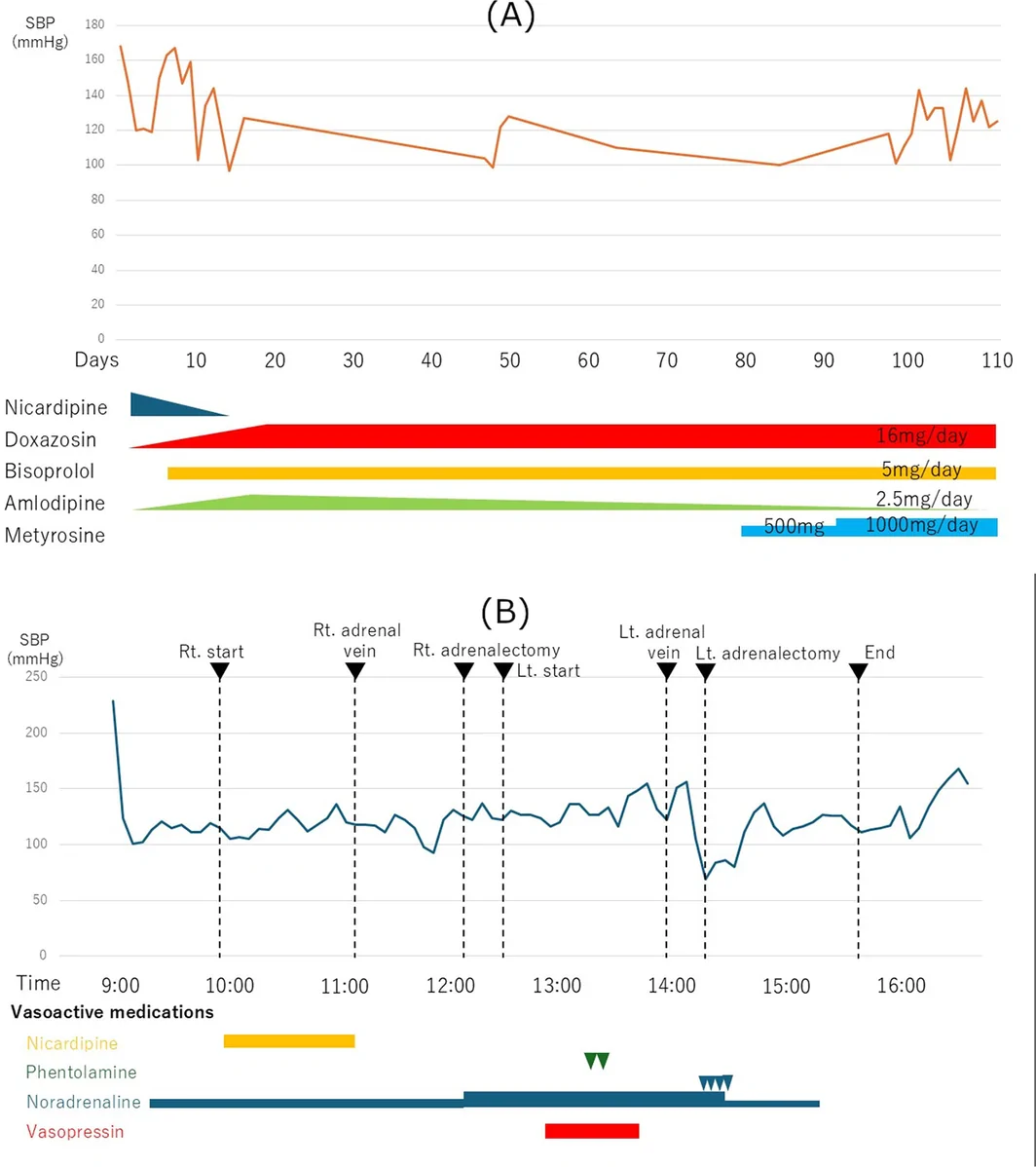
Perioperative clinical course. Changes in blood pressure and antihypertensive medications from admission to surgery. Intraoperative blood pressure changes and vasoactive medications during bilateral adrenalectomy.

Because of the preceding life‐threatening crisis, severe cardiac dysfunction, marked catecholamine excess, bilateral disease, and large left‐sided tumor, the patient was considered at high perioperative risk. Metyrosine was added to α‐adrenergic blockade at 500 mg/day 2 weeks before surgery and increased to 1000 mg/day 1 week later. Doxazosin was titrated to 16 mg/day. Blood pressure remained stable without symptomatic hypotension (Figure 3A), while total urinary metanephrine decreased from 20.4 to 4.86 mg/day. Only mild somnolence occurred, and LVEF improved to 64.8%.

On Day 107, one‐stage bilateral total adrenalectomy was performed. Cortical‐sparing adrenalectomy was considered, but only limited normal adrenal parenchyma was identifiable on imaging. The 4.9‐cm right tumor was removed laparoscopically, whereas the 7.7‐cm left tumor was removed through an open Chevron incision. Transient hypertension occurred during anesthesia induction and tumor manipulation, followed by hypotension requiring temporary vasopressors after tumor removal (Figure 3B). Postoperatively, blood pressure remained stable, and steroid replacement was initiated. Histopathology confirmed bilateral pheochromocytomas. Postoperative RET testing identified a c.1901G>A (p.Cys634Tyr) mutation, establishing the diagnosis of MEN2A.

## 3 Discussion

Pheochromocytoma crisis is a life‐threatening condition caused by excessive catecholamine release and may result in cardiovascular dysfunction, respiratory failure, and multiorgan failure [1]. Pheochromocytoma‐associated cardiomyopathy occurs more frequently in younger patients than conventional Takotsubo syndrome and may present with atypical patterns, including reverse Takotsubo cardiomyopathy [2]. In the present case, severe cardiac dysfunction and respiratory failure were consistent with systemic catecholamine toxicity.

The patient was considered at high perioperative risk because of several overlapping factors, including a preceding life‐threatening pheochromocytoma crisis, severe cardiac dysfunction, marked catecholamine excess, bilateral disease, and a large left‐sided tumor. Although α‐adrenergic blockade remains standard preoperative treatment, metyrosine may be added in selected high‐risk patients to inhibit catecholamine synthesis [3, 4]. Given these risk factors and the planned one‐stage bilateral adrenalectomy, metyrosine was added to α‐adrenergic blockade.

The optimal dose and duration of preoperative metyrosine treatment have not been established, although doses up to 4000 mg/day have been used [4]. A longer treatment period with further dose escalation was initially considered. However, blood pressure was already adequately controlled, and antihypertensive medication reduction was being considered. Because further catecholamine suppression might contribute to excessive hypotension, metyrosine was cautiously administered at 500 mg/day followed by 1000 mg/day 1 week later. Total urinary metanephrine decreased by approximately 76% without symptomatic hypotension, and only mild somnolence occurred. Given this response, surgery was performed without further dose escalation.

Nevertheless, transient hypertension occurred during anesthesia induction and tumor manipulation, followed by hypotension requiring vasopressors after tumor removal. Thus, metyrosine did not eliminate the characteristic hemodynamic fluctuations of pheochromocytoma surgery. Whether a higher dose, longer duration, or different dose‐escalation strategy would have provided greater hemodynamic benefit remains uncertain. However, the marked biochemical suppression without serious adverse events suggests that cautious metyrosine administration may be feasible in selected high‐risk patients.

Bilateral pheochromocytomas and medullary thyroid carcinoma strongly suggested MEN2A, although RET testing performed preoperatively was not available at surgery [5, 6]. Cortical‐sparing adrenalectomy may preserve adrenal function in MEN2‐associated bilateral pheochromocytomas, although steroid dependence and recurrence remain concerns [7]. In this case, only limited normal adrenal parenchyma was identifiable on imaging. Moreover, extensive tumor manipulation to preserve the small amount of residual cortex was considered to risk hemodynamic instability. MEN2A had not yet been genetically confirmed, and malignant potential could not be excluded, particularly for the large left‐sided tumor. Bilateral total adrenalectomy was therefore selected despite the need for lifelong steroid replacement. Postoperative RET testing confirmed MEN2A.

The surgical approach was individualized according to tumor size and anatomy. The smaller right‐sided tumor was approached laparoscopically, allowing exposure by liver mobilization, whereas the larger left‐sided tumor was removed through an open Chevron incision to ensure adequate exposure and tumor extraction.

## 4 Conclusion

Preoperative metyrosine administration achieved marked biochemical suppression of catecholamine production without clinically significant adverse effects in a high‐risk patient with bilateral pheochromocytomas. Although its effect on intraoperative hemodynamic stability remains uncertain, this case supports the feasibility of metyrosine as an adjunct to conventional α‐adrenergic blockade in carefully selected patients.

## Ethics Statement

The authors have nothing to report.

## Consent

Written informed consent for publication was obtained from the patient.

## Conflicts of Interest

The authors declare no conflicts of interest.

## Data Availability

The data that support the findings of this study are available on request from the corresponding author. The data are not publicly available due to privacy or ethical restrictions.
